# Effect of posterior gingival smile on the perception of smile esthetics

**DOI:** 10.4317/medoral.19167

**Published:** 2013-08-29

**Authors:** Alicia Rodríguez-Martínez, Ascensión Vicente-Hernández, Luis A. Bravo-González

**Affiliations:** 1 DDS, Master in Orthodontics, Murcia, Spain; 2 DDS, PhD, Contracted Doctor, Orthodontics Teaching Unit, University Dental Clinic, Faculty of Medicine and Dentistry, University of Murcia, Murcia, Spain; 3 MD, DDS, MS, PhD, Senior Lecturer, Orthodontics Teaching Unit, University Dental Clinic, Faculty of Medicine and Dentistry, University of Murcia, Murcia, Spain

## Abstract

Objectives: To evaluate and compare the influence of posterior gummy smile on the perception of smile esthetics by orthodontists, general-dentists and laypersons. Study Design: A frontal photograph of a smile with normal gum exposure was chosen and manipulated digitally using Adobe Photoshop C3 to generate three further images with posterior gum exposure of 4, 6 and 8mm. These four images were assessed by the three evaluator groups: orthodontists (n=40), general-dentists (n=40) and laypersons (n=40). Both orthodontists and dentists had at least ten years professional experience and laypersons were aged between 40-50 years. The proportion of men to women was 20:20 in each group. Evaluators awarded a score to the smile esthetics of each image: 1=acceptable, 2=moderately acceptable, 3=unacceptable. Afterwards, each evaluator placed the four images in order of esthetic preference. Results: No significant differences (p>0.05) were detected between the three evaluator groups for the photo without posterior gummy smile. The perception of smile esthetics for a the 4mm posterior gummy smile (median for orthodontists=2, general-dentists= 1, laypersons=1), the 6mm (median for orthodontists=2, general-dentists=1, laypersons=1) and the 8mm (median for orthodontists=3, general-dentists=2, laypersons=2) was significantly different between orthodontists and the other two evaluator groups (p<0.0017). The three evaluator groups coincided in placing the image with the 6mm gum exposure in first place in order of esthetic preference. Conclusions: Posterior gummy smile influences the perception of smile esthetics more negatively among orthodontists than the rest of the groups.

** Key words:**Aesthetics, gummy smile back, orthodontists, dentists, laypersons.

## Introduction

The criteria by which esthetics are determined to be acceptable or not vary according to social and cultural conventions, and these have been subject to change throughout history. In the present era, the esthetic parameters not only of the face and body but also of the smile have become important issues.

Smile esthetics have been widely studied in the field of orthodontics. The attractiveness of a face depends on a range of features and arrangements of which the eyes and the smile are among the most important (1-3). Beall (4) claims that individuals with attractive teeth and harmonious smiles are considered more attractive, more intelligent and more popular than those that do not possess these attributes.

There are a number of characteristics that must be considered when it comes to assessing whether or not a smile is harmonious. Among these is the presence or absence of what is known as gummy smile.

Anterior gummy smile has been widely studied throughout the history of orthodontics; its etiology (5), treatment and impact on esthetics as perceived by different sectors of the population have been widely analyzed (6-8). These studies have made it possible to establish certain criteria with regard to the amount of gum exposure considered esthetically acceptable. Morley et al. (9) opted for a range of 1-3 mm anterior gum exposure, while Kokich et al. (6) put forward a maximum gum exposure of 3 mm as the esthetic norm, and Geron et al. (10) put forward an upper limit of 1 mm.

However, although posterior gummy smile also plays an important part in perceived smile esthetics, to date no evaluation of the amount of gum exposure that might be considered an esthetic norm has been established. As far as we are aware, no research has been published that evaluates the esthetic impact of this feature across different groups of evaluators.

For this reason, the aim of this study was to determine the influence of posterior gummy smile on perceived smile esthetics by orthodontists, general dentists and lay persons.

## Material and Methods

Three groups of evaluators took part in the study: orthodontists, general dentists and lay persons. Each group was made up of 40 individuals with a proportion of 20 men to 20 women.

Both orthodontists and dentists had all been in professional practice for over ten years. Lay persons were all aged between 40 and 50 years.

An individual was selected from among patients attending the University Dental Clinic with 0º posterior gum exposure. This patient gave informed consent to allow a photograph of his smile to be digitally manipulated for use in the study.

A color photograph was taken frontally, with the subject posed in a natural head position, using a digital camera (Canon EOS 450 D, Madrid, Spain). The original photo was then manipulated digitally using Adobe Photoshop CS3 (Adobe, Systems Inc. San José, California). The original had a smile with 0 mm gingival exposure, this was modified digitally to generate three further images with 4 mm, 6 mm and 8 mm posterior gum exposure. The photos were cropped to exclude the nose and chin.

Catalogue

**Figure 1:**
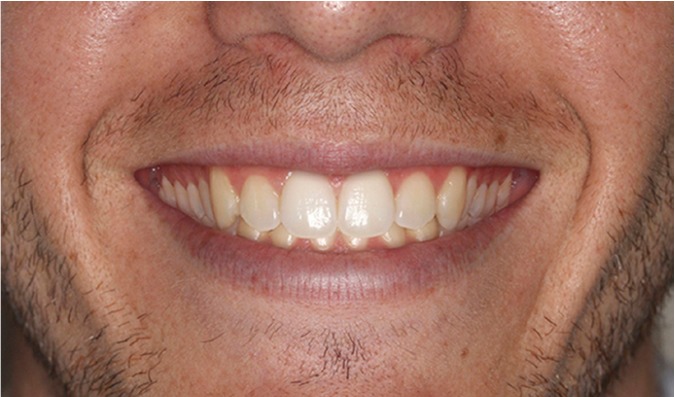
Photo of smile modified to create 4 mm of posterior gum exposure.

**Figure 2:**
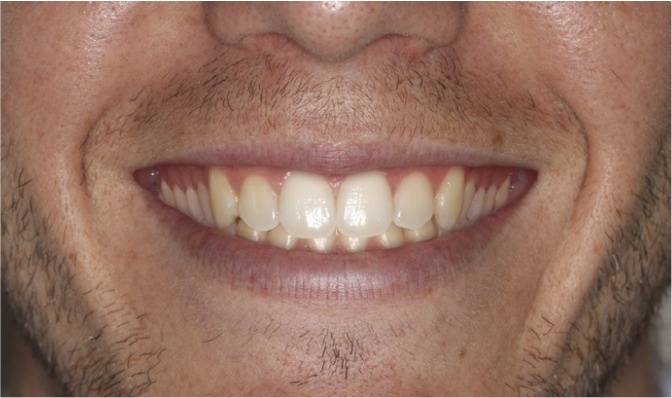
Photo of smile modified to create 6 mm of posterior gum exposure.

**Figure 3:**
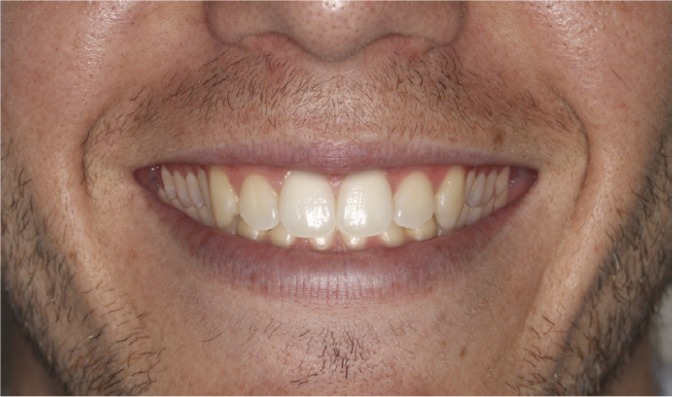
Photo of original smile with 0 mm of posterior gum exposure.

**Figure 4:**
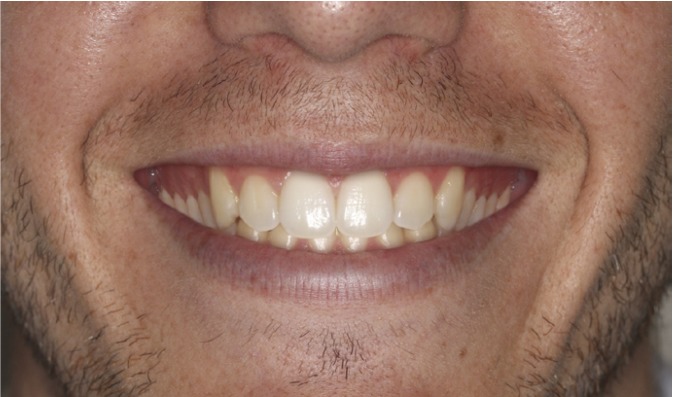
Photo of smile modified to create 8 mm of posterior gum exposure.

The four images were presented to the evaluators as a catalogue. This was organized so that the first page showed the 4 mm gummy smile (Fig. 1), followed by 6 mm (Fig. 2), 0 mm (Fig. 3) and lastly the 8 mm gum exposure (Fig. 4). The fifth page of the catalogue showed all four images together.

Evaluators were asked to view each page for no more than 40 seconds, after which they filled out a questionnaire. They were not permitted to look back at previously viewed images.

Questionnaire

A questionnaire collected details of which evaluator group each subject belonged to – orthodontists, dentists or lay persons – as well as sex, age and, in the case of orthodontists and dentists, years of professional experience.

Each catalogue image was evaluated using a point scale as follows: 1=esthetically acceptable, 2=moderately acceptable, 3=esthetically unacceptable. The questionnaire also asked the evaluators to place the four images appearing together on the fifth page in order of esthetic preference from most favorable to the least.

The use of the questionnaire was supervised by a member of the research team who ensured that the images were not viewed for more than 40 seconds and that evaluators did not turn back to look at previous images. The same staff member filled out the questionnaire registering the evaluators’ responses.

Method Error

Ten subjects from each group completed the questionnaire again after a two-week interval. The Wilcoxon Test for paired samples did not detect significant differences between image evaluations performed at these two different times (p>0.05). Statistically significant differences in the results obtained between the two evaluations were not detected. Orthodontists: (Fig. 1), p=0.56;( Fig. 2), p=1; (Fig. 3), p=0.33;( Fig. 4), p=1. Dentists: (Fig. 1), p=1; (Fig. 2), p=0.75; (Fig. 3), p=0.18; ( Fig. 4), p=0.31. Lay persons: (Fig. 1), p=0.157; (Fig. 2), p=0.31; (Fig. 3), p=1; (Fig. 4), p=0.58. Order of preference p=0.28). With regard to the order of preference, when data from the two time points were analyzed, orthodontists coincided across the two evaluation times in 100% of cases, while lay persons and dentists in 70% of cases.

Statistical Analysis

For individual image evaluations, significant differences between the three groups were analyzed using the Kruskal-Wallis (p<0.05) and the Mann-Whitney test applying the Bonferroni Correction (p<0.0017).

Order of esthetic preference allotted to each image by the three evaluator groups was analyzed with the Kruskal-Wallis non-parametric test (p<0.05).

In the aim of determining the presence of significant differences between groups for the order of esthetic preference awarded to each image, the Friedman test was applied (p<0.05). Post hoc analysis was performed using the Wilcoxon test for paired samples checking significance with the Bonferroni correction (p<0.008).

Afterwards, in order to analyze which image had more statistical dispersion in the order of preference, squared standard deviations from the mean were calculated for all preferences and the existence of significant differences was determined using the Kruskal-Wallis and the Mann-Whitney tests applying the Bonferroni Correction (p<0.008).

## Results

**Figure 5:**
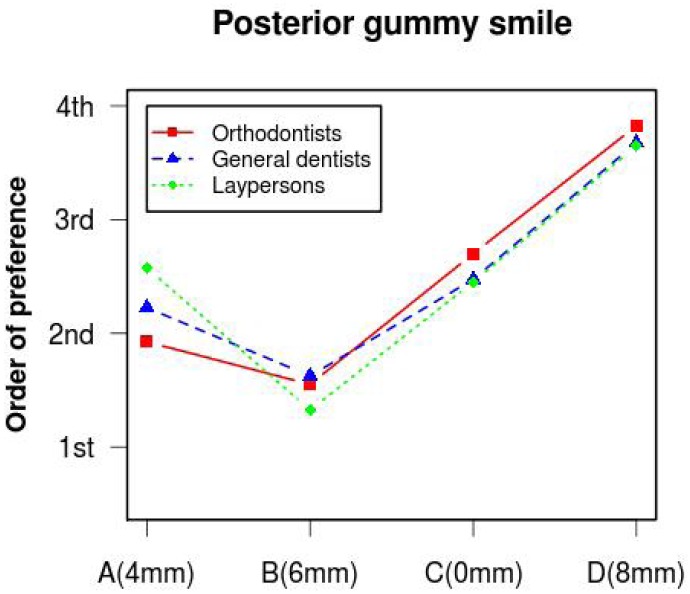
Order of preference given for each photograph (A= Fig. 1; B= Fig. 2; C= Fig. 3 and D= Fig. 4) by the three groups of evaluators.

The Kruskal-Wallis test did not detect significant differences (p>0.05) between the three evaluator groups in their assessment of (Fig. 3) (0 mm). Analysis of the esthetic perception of (Fig. 1) (4 mm), (Fig. 2) (6 mm) and (Fig. 4) (8 mm) revealed significant differences between orthodontists and the other evaluator groups (p<0.0017). ( Table 1) (Fig. 5) shows that, when placing the images in order of esthetic preference, (Fig. 2) (6 mm) was considered the most acceptable by all three groups.

**Table 1:**
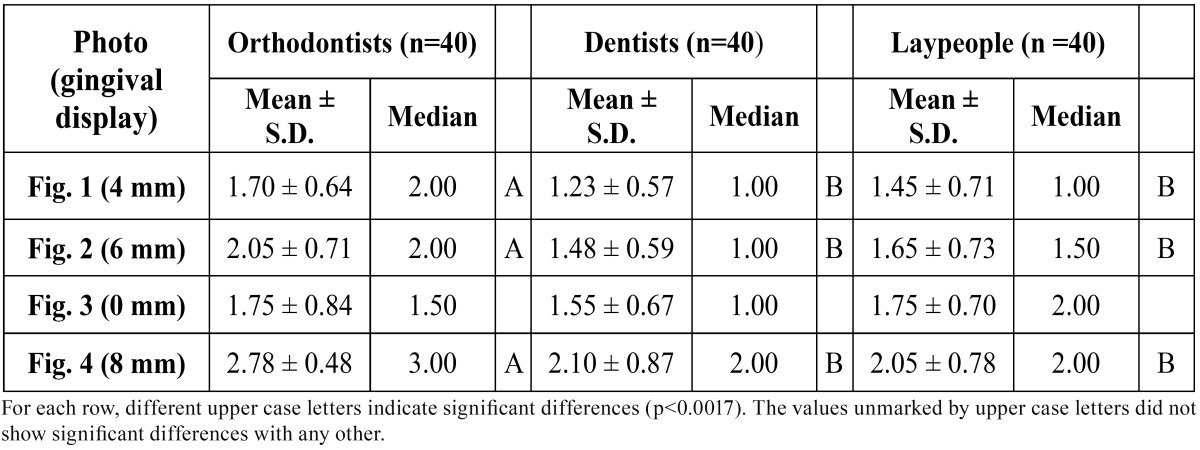
Mean, Standard deviation (S.D.) and median for attractiveness scores of the photographs with different amount of gingival display.

The Kruskal-Wallis test did not detect significant differences in the order of preference awarded to each image by the three evaluator groups (p>0.05). ( Table 2).

**Table 2:**
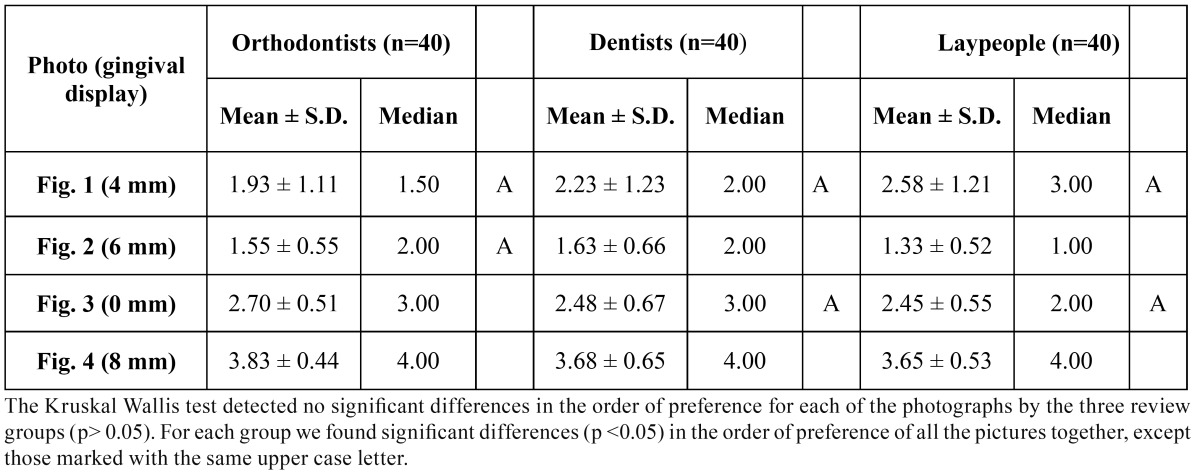
Mean and Standard deviation (S.D.) for the order of preference of each photograph.

The Friedman test indicated significant differences (p<0.05) in the order of preference allotted to each image within each evaluator group. The Wilcoxon test showed for orthodontists significant differences in all comparisons except for order of preference, of (Fig. 1) (4mm) and (Fig. 2) (6mm). Meanwhile for dentists and laypersons there were significant differences (p<0.05) in all comparisons except between (Fig. 3) (0mm) and (Fig. 1) (4mm) (Table 2).

When squared standard deviations from the mean were analyzed by means of the Kruskal-Wallis test, significant differences (p<0.05) were found and the Mann-Whitney test determined that the squared standard deviation from the mean for (Fig. 1) (4 mm) was significantly greater than those of the rest of the images (p<0.008) ( Table 3).

**Table 3:**
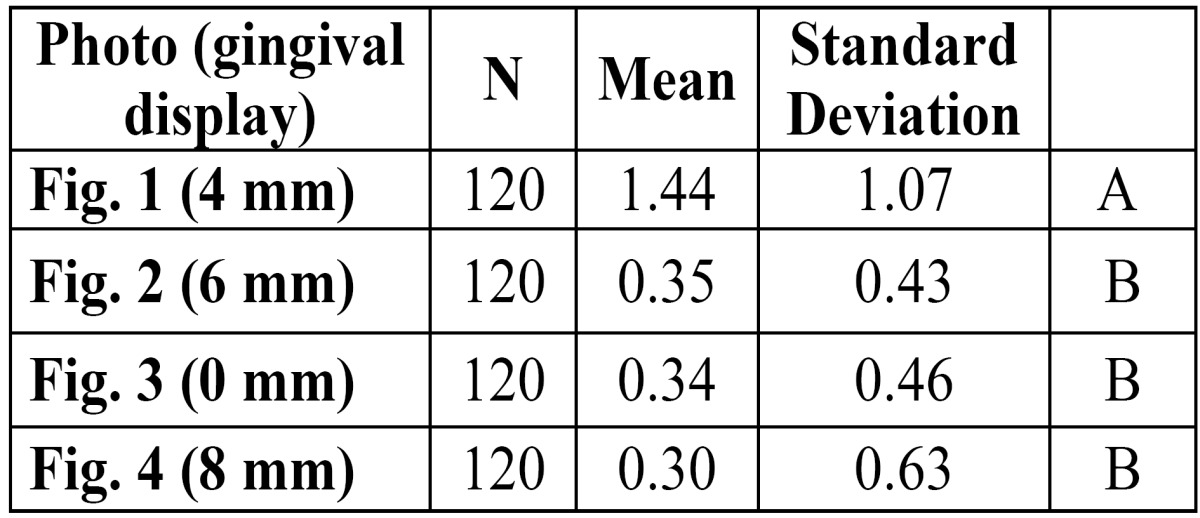
Squared deviations from the mean of each photograph. Different upper case letters indicate significant differences (p<0.008).

## Discussion

This study analyzes evaluations of posterior gummy smile made by three population groups: orthodontists, general dentists and lay persons. The objective was to determine how and to what extent professional practice affected the perception of posterior gummy smile esthetics, given that various authors have shown that the profession of an evaluator markedly influences the perception of smile esthetics (6,11,12).

In order to homogenize the sample of evaluators, inclusion criteria were established whereby all evaluators were aged between 40 and 50 years and the proportion of men and women was the same in each group.

In order to reduce the effects of confounding factors that a complete facial image may create (6,13,14), the images used in the study were cropped so that they only showed the subject’s mouth.

Similar to the study by Alahija et al. (11), the smile images were evaluated awarding different scores: 1=esthetically acceptable, 2=moderately acceptable, 3=esthetically unacceptable. Other researchers have used visual analogue scales (VAS) for judging the attractiveness of a smile (6,14-17). Nevertheless, the present study opted for a points system, as the literature affirms that this method provides results that are simpler, quicker and more reproducible than a VAS.

The results of this study showed that all three evaluator groups made similar assessments of (Fig. 3) (0 mm), and these were between esthetically acceptable and moderately acceptable.

As for (Fig. 1) (4 mm), (Fig. 2) (6 mm) and (Fig. 4) (8 mm) the evaluations made by dentists and lay persons were similar. However, the opinion of orthodontists was significantly different from the other two groups. While orthodontists found (Fig. 1) (4mm) and (Fig. 2) (6mm) only moderately acceptable, dentists and lay persons thought them esthetically acceptable. (Fig. 4) (8mm) was considered esthetically unacceptable by orthodontists but moderately acceptable by dentists and lay persons. These data highlight the fact that orthodontists had a more critical perception of posterior gummy smile than the other two groups – the greater the extent of posterior gum exposure the more negative the evaluation by orthodontists compared to dentists and lay persons. This critical trait among orthodontists has been evidenced in other studies of anterior gummy smile such as that of Kokich et al. (18) who showed that orthodontists establish a maximum esthetic limit to anterior gum exposure of 2 mm, while the general public put this at 4 mm. There are other studies (11,19) in which it was observed that laypersons were less critical than dentists and orthodontists, in other words dental professionals in general, who applied similar esthetic criteria.

When the three evaluator groups set about placing the images in order of esthetic preference, all coincided in showing preference for the 6 mm image. In this way, when all images were showed together, all three groups considered it more esthetically pleasing to expose a certain extent of posterior gum. However, while orthodontists gave a similar order of preference to (Fig. 1) (4 mm) and (Fig. 2) (6 mm), dentists and lay persons did not discern a difference in order of preference between (Fig. 3) (0 mm) and (Fig. 1) (4 mm). This shows again that orthodontists have a higher level of clinical perception than dentists or lay persons, as orthodontists perceived images with 2 mm difference in gum exposure as similar, while dentists and lay persons saw images with a difference in gum exposure of 4 mm as similar.

Furthermore, the method error test found that the decisions as to order of preference among orthodontists coincided 100% over the two time points, while dentists and laypersons coincided in only 70% of cases.

(Fig. 1) (4 mm) represented gummy smile esthetics that provoked the least agreement among the evaluators when it came to placing it in order of preference. This image showed an intermediate posterior gum exposure and this caused the greatest variation in awarding preference.

On the basis of these results, it might be established that:

1.- Posterior gummy smile had a more negative influence in the perception of smile esthetics among orthodontists than dentists and lay persons. There were no statistically significant differences in evaluations of posterior gummy smile between general dentists and lay persons.

2.- In the sequence of images all groups showed preference for the image with 6 mm of posterior gum exposure.

3.- Orthodontists gave the same order of preference to images with 4 and 6 mm of posterior gum exposure, while dentists and lay persons did the same for images with 0 and 4 mm of posterior gum exposure.

4.- The image that showed the most variation when it came to placing it in order of esthetic preference was (Fig. 1) (4mm).

5.- In general, until 6 mm of posterior gum exposure was considered esthetically acceptable.
